# Utilization of a Continuous External Tissue Expansion System to Assist in Primary Closure of a Large Anterolateral Thigh Donor Site Defect

**DOI:** 10.1155/2014/860749

**Published:** 2014-03-25

**Authors:** Andrew G. Silver, Richard C. Baynosa

**Affiliations:** Division of Plastic Surgery, University of Nevada School of Medicine, 2040 West Charleston Boulevard, Suite 301, Las Vegas, NV 89102, USA

## Abstract

Primary closure of a large anterolateral thigh (ALT) flap donor site defect with the assistance of an external tissue expansion system is presented. The dimensions of this donor site (12 cm × 40 cm) and its percentage of leg circumference (34%) would make this site likely to require skin grafting or further flap coverage based on the results of previously published literature.

## 1. Introduction

The anterolateral thigh (ALT) flap has become a popular choice of reconstructive surgeons when soft tissues are required for a given defect. Although the donor site morbidity is generally thought to be minimal, a significant cosmetic deformity can result, especially when a skin graft is required for closure of larger donor sites [1–3]. Additional reported complications of the utilization of skin grafts at ALT donor sites include the following: lack of durability, pain, contour deformity, and limited maneuverability from adhesions to underlying musculature [1–6]. Although these complications can essentially be negated through primary closure of the donor site, compartment syndrome has been reported as a complication of overly aggressive closure [6].

With regard to potential primary closure of the ALT donor site, several studies have been published to elucidate which defects are likely to necessitate a skin graft for closure. In their experience with 672 ALT flaps, Wei et al. stated that 40% of their donor sites, often those with width greater than 6 to 9 cm, would require a skin graft [7]. Perhaps Boca et al., who found that a flap width greater than 16% of the midthigh circumference was likely to require a skin graft, have determined a superior preoperative indicator [8].

In order to facilitate the closure of the donor site without the need for a skin graft, several techniques have been described. These techniques include additional flaps and internal tissue expansion, the placement of which has been described before, during, and after the time of flap harvest [9, 10]. To the authors knowledge, there have not been any published reports of the utilization of an external tissue expander in the closure of these donor sites.

## 2. Case Report

**Figure 1:**
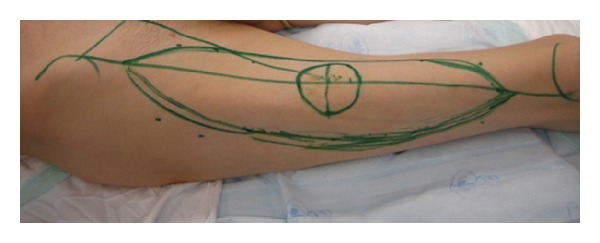
ALT flap design. Dimensions 12 cm × 40 cm.

The patient was a 55-year-old diabetic male who developed necrotizing fasciitis of the left foot and leg. After the infection was eradicated, a large soft tissue deficit was present. This deficit was reconstructed with an ALT flap (design in Figure 1) with a length of 40 cm and a width of 12 cm. The circumference of the thigh at the midpoint of the flap was 35 cm.

The tissue lateral and medial to the defect was extensively undermined. The superficial fascia was closed with 2-0 absorbable sutures in simple interrupted fashion. The cephalad and caudal most aspects of the donor site were easily reapproximated with 3-0 absorbable sutures placed in the deep dermal layer followed by staple closure of the epidermis.

**Figure 2:**
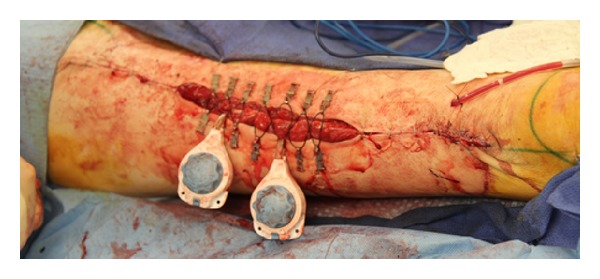
External tissue expanders applied to aid in closure of donor site where approximation with staples was not possible.

**Figure 3:**
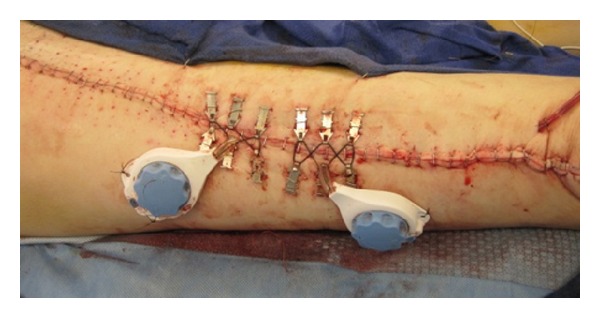
Primary closure obtained with utilization of two Dermaclose systems.

The remainder of the defect was not suitable for primary closure utilizing this method. A continuous external tissue expansion system (DermaClose, Wound Care Technologies, Chanhassen, MN) was then applied in the shoelace technique with six anchors as described in the product insert at the cranial aspect of the remaining defect. Mechanical creep could be appreciated within minutes, which then allowed for primary closure in this portion of the wound. The expansion system was then removed and reapplied in a caudal direction and the process was repeated. Simultaneously, an additional external tissue expansion system was utilized in a caudal to cranial fashion until the wound was closed with the expanders meeting in the middle of the incision (Figures 2 and 3). The entirety of the wound was closed primarily at the initial procedure. A drain was left in place given the extensive undermining.

**Figure 4:**
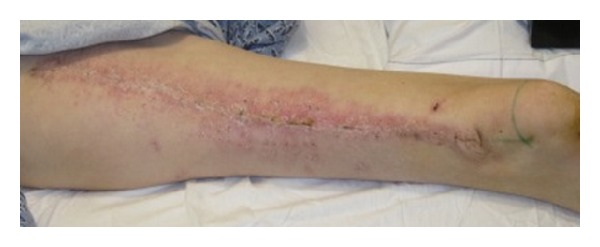
One month postoperative appearance.

The external tissue expanders were left in place for one week postoperatively to reduce tension on the closure and to provide further expansion. During this time, the patient experienced a sensation of tightness in the thigh but had no other complaints in regard to the donor site. The expanders were then removed at the bedside with only one dose of intravenous morphine required for the comfort of the patient. The appearance of the donor site at one month postoperatively is shown in Figure 4. The donor site has now been stable in this appearance for a total of four months.

## 3. Discussion

We have described the utilization of an external tissue expansion system to aid in the primary closure of a large ALT donor site. According to the aforementioned published literature, the dimensions of this donor site (12 cm × 40 cm) would make it likely to require a skin graft or an additional flap procedure in order to achieve primary closure. Further, the width of this flap was 34% of the circumference of the thigh, which by the work of Boca et al. would also make the site unlikely to be amendable to primary closure.

Skin grafting of the ALT donor site is a possible means of wound coverage that has several potential drawbacks, including but not limited to poor durability, contour irregularity, and the necessity of graft harvesting with resultant scarring.

When compared to skin grafting, additional flap coverage of the donor site provides more durable tissue and lessens contour irregularity, yet the requisite of an additional donor site and any associated complications are not eliminated. To obviate the creation of an additional donor site, internal tissue expanders have been utilized at all stages of reconstruction; however, this involves at the very least a second procedure. Applying an external tissue expansion system that provided intraoperative expansion and continuous postoperative tension relief at the suture line, we were able to close this large donor site primarily, without sacrificing any additional tissue. Removal of the devices was performed at the bedside one week postoperatively, and coverage has been stable at a follow-up time of six months.
